# Fertility With Early Reduction of Ovarian Reserve

**DOI:** 10.7759/cureus.30326

**Published:** 2022-10-15

**Authors:** Dipanshu K Kesharwani, Shazia Mohammad, Neema Acharya, Ketav S Joshi

**Affiliations:** 1 Obstetrics and Gynaecology, Jawaharlal Nehru Medical College, Datta Meghe Institute of Medical Sciences (Deemed to be University), Wardha, IND; 2 Department of Obstetrics and Gynaecology, Jawaharlal Nehru Medical College, Datta Meghe Institute of Medical Sciences (Deemed to be University), Wardha, IND

**Keywords:** antral follicle count (a.f.c.), f.s.h, antimullerian hormone (a.m.h.), p.o.r, poor ovarian reserve, dor, diminished ovarian reserve, infertility

## Abstract

Female infertility is defined as the failure to conceive after a year of frequent, unprotected sexual activity. Infertility affects 8-10% of females worldwide. There are many causes of infertility. One of them is diminished ovarian reserve (DOR). In this condition, the ovary loses its reproductive potential, which affects fertility. This condition may be caused due to injury, but it usually results from aging. DOR is one of the main reasons for infertility in women worldwide. A woman with DOR has fewer eggs in her ovaries than usual. The quality of the remaining eggs may not be bad. This condition impairs the development of existing eggs. Patients with DOR may be able to get pregnant if they are properly treated according to their profile. Their treatments are individually tailored according to their needs. These patients should be recommended a robust approach toward treatment and increasing fertility. The chances of pregnancy increase if the treatment is started early.

## Introduction and background

There is scarce knowledge regarding the dangers and treatment of diminished ovarian reserve (DOR). However, we know that DOR is influenced by aging, genetics, and the environment [1,2]. We are also confident that DOR is irreversible and that ovarian stimulation in assisted reproductive technologies (ART) works effectively for women with DOR. DOR decreases the quality and number of oocytes, a bad prognostic factor for ART [3]. Several factors lead to DOR, such as age, stress, lifestyle, etc. Of all these factors, age is the most common cause that leads to DOR [3]. Aberrant vascularization, free radical imbalance, and toxic and genetic changes all hamper the condition and quantity of oocytes, leading to atypical fertilization and abnormal embryo implantation. Increased cycle cancellation rates and lower pregnancy rates have all been associated with DOR while performing in vitro fertilization (IVF) [4].

Definition

DOR is characterized by a decline in the volume and quality of oocytes [5]. It can be used to define reproductive-age women with periodic, primarily ovulatory cycles with lower fecundity or stimulation responses than other women their age. It is different from early ovarian insufficiency or menopause. Other factors outside a woman's chronological age affect the oocyte count in her ovarian reserve. Recent studies show that the cause of DOR and the success of IVF are related.

Ovarian stimulation in IVF may result in an abnormally low follicular response, which means that fewer eggs are extracted from the ovary [4]. This is known as poor ovarian response (POR). According to the European Society of Human Reproduction and Embryology (ESHRE), the Bologna criteria may be used to define POR. To standardize the definition, the Bologna criteria are utilized.

The Bologna criteria are presented in Table 1 [6].

**Table 1:** Bologna criteria POR: poor ovarian reserve; FSH: follicle-stimulating hormone; AFC: antral follicle count; AMH: anti-müllerian hormone

Criteria (any two of the following)	Parameters
Advanced maternal age or any other risk factors for POR	>40 years of age
Previous history of POR	≤3 oocytes with conventional stimulation of >149 IU FSH daily
Low ovarian reserve test	AFC <5-7, or AMH <0.5-1.1 ng/ml

OR Following maximum ovarian stimulation, two episodes of POR are enough to identify a patient as a poor responder.

DOR is the most common cause of POR. Specific subgroups of DOR have better IVF outcomes than others, depending on the cause of DOR. A recent case-control study found that DOR brought on by endometrioma surgery responded better to IVF compared to the idiopathic group [7].

## Review

Causes of DOR

DOR is caused by several factors, as depicted in Table 2.

**Table 2:** Causes of DOR DOR: diminished ovarian reserve

Causes	
Idiopathic	Like accelerated oocyte apoptosis
Chemotherapy	Depletes primordial follicles [8,9,10]
Radiotherapy	Affects the ovaries according to the dosage and the patient's age.
Genetic mutations	Like FMR
Lifestyle	Like tobacco consumption, smoking
Surgeries	Ovarian surgeries, tubal surgeries
Infections	Like mumps
Autoimmunity	Like Addison's disease, lymphocytic oophoritis, celiac disease, Polyglandular syndrome, Hashimoto's thyroiditis
Metabolic disorder	Like galactosemia

According to Barker's hypothesis, hormonal imbalance during pregnancy may cause DOR in the fetus if the fetus is female [11].

Estimation of ovarian reserve

Ovarian reserve can be defined as the quality and quantity of oocytes in a woman's ovaries, indicating the woman's ability to conceive. Several tests are employed nowadays to estimate ovarian reserve, called ovarian reserve tests (ORT). ORT helps estimate ovarian reserve, measure the woman's reproductive life, and predict menopausal timing so that women can start planning for a family. It also helps to see the response to ovarian stimulation. There is a misconception regarding ovarian reserve that a woman cannot get pregnant if ORT shows low ovarian reserve. While It is difficult to get pregnant if the ovarian reserve is low, but not entirely impossible. A woman should undergo a battery of ORTs for a proper diagnosis and to know her reproductive potential [12].

ORTs should not be too expensive and should be available to most of the population. It should be preferably non-invasive or minimally invasive with high sensitivity and specificity. It should identify a decrease in ovarian reserve at the initial stages so that appropriate medication and treatment may be initiated to aid the patient in conceiving [13].

They are different types of ORTs, such as provocative, biochemical, and sonographic images of ovaries. Hormonal markers and ultrasound images help estimate the number of oocytes in ovaries and predict the chances of the patient getting pregnant naturally or via ART [14]. These markers and parameters include the concentration of follicle-stimulating hormone (FSH), luteinizing hormone (LH), anti-müllerian hormone (AMH), and antral follicle count (AFC). Age is a significant factor when it comes to these tests. In light of new research, dynamic tests to evaluate ovarian function have been developed. These tests use a GnRH agonist, FSH, or clomiphene citrate.

Hormonal markers

Basal FSH and Estradiol

Basal FSH testing has undergone extensive research and has been shown to be an easy and accurate procedure. It is currently the most common screening test used in infertility programs because it is more affordable than the other tests and is simple to use. The decline in ovarian follicles is followed by an increase in FSH production by the pituitary. In women undergoing IVF, early follicular FSH and age are more effective at predicting outcomes than age alone (IVF) [2]. The inability to determine a precise cutoff point, monthly changes, and discrepancies between various laboratory tests are all limitations of basal FSH monitoring.

Lower basal FSH and estradiol (E2) are associated with better pregnancy rates and ovarian reserve during IVF utilizing a GnRH antagonist. However, LH levels did not drop compared to FSH and estradiol. In women with ovarian failure, characterized by excessive early follicular FSH and irregular menstrual cycles, the average LH amplitude, LH response to GnRH, and LH concentration increased [15,16].

Additionally, the patients who undergo ovarian stimulation during IVF can use the FSH:LH ratio to predict their chances of getting pregnant. A higher basal FSH:LH ratio implies diminished ovarian reserve even with an average FSH level. If FSH:LH level is high and associated with an average early follicular FSH, the body may respond poorly to controlled ovarian hyperstimulation [17].

There is an inverse relationship between the basal level of estradiol and ovarian response. Early follicular estradiol works more efficiently with basal FSH to estimate ovarian reserve than estradiol alone. High basal estradiol and normal FSH are linked with increased cycle cancellation rates and indicate poor ovarian response [18].

Anti-müllerian Hormone

Granulosa cells in growing preantral and small antral follicles secrete anti-müllerian hormone (AMH). The level of AMH reaches its peak at the age of 25 years and then starts declining gradually until it becomes undetectable a few years before menopause [19,20]. AMH is mostly a gonadotropin-independent hormone secreted during the early follicular stage. Recent studies have shown that AMH levels do not vary much; if they do change, they are usually younger [21].

AMH is a sensitive biomarker of ovarian reserve as it declines before FSH rises. In addition to effectively predicting the date of menopause, this test demonstrates an excellent relationship between the primordial follicle pool and ovarian response to ovarian stimulation. Analysis of serum AMH levels aids in ovarian age evaluation, ovulation induction response prediction prior to IVF, risk assessment of ovarian hyperstimulation syndrome, identification of polycystic ovarian syndrome (PCOS), and monitoring therapy response.

Enzyme-linked immunosorbent assay (ELISA) Gen II is used to evaluate AMH [22]. Compared to traditional ELISA tests, newer automated AMH assay systems reportedly offer improved accuracy, sensitivity, and quicker findings. Several things commonly affect AMH levels, such as racial background and nationality, surgery for ovaries, smoking addiction, vitamin D deficiency, PCOS, and suppression of ovaries due to oral contraceptive pill (OCP) and PCOS [23]. AMH may have the following benefits over other traditional ovarian reserve markers: (1) it changes the earliest with age; (2) it also shows minimum variability between two consecutive cycles; (3) it has the minimum variability during a single cycle; and (4) it could be helpful if taken at random during the cycle.

However, there are certain limitations to considering AMH as a biomarker for infertility as recent studies have shown that heterogeneity exists in AMH trajectories, which may hamper application in personalized patient counseling. Since there is no international standard for AMH to establish assay-independent cutoff values, we cannot use AMH as an indicator for infertility.

Antral Follicle Count (AFC)

AFC is the total amount of follicles in both ovaries on sonography taken between two to four days of the early follicular phase. Antral follicles can be defined as those with a two-dimensional diameter of between 2 and 10 mm. AFC is simple to perform and provides faster results. This does not show much variability when performed by different observers and when performed on multiple cycles [24]. However, precision is affected when performed on women of extreme weights and intra-cycle dependency. According to a study by Broekmans et al. on ovarian reserve testing and IVF outcomes, low AFC is linked to POR but has a low pregnancy probability [24]. AFC's specificity for predicting non-pregnancy varies from 64% to 98%, while its sensitivity remains poor at 7% to 34%. Since it also assesses atretic follicles of the same size, AFC exaggerates the number of oocyte and FSH-sensitive follicles [25].

Ultrasound

It has been demonstrated that ovarian reserve is associated with sonographic assessments of the volume of ovaries and antral follicles in the follicular phase. Observed cycle differences, biological variance, and intra-observer discrepancies are certain issues associated with AFC [26]. Additionally, it has been proposed that pulsed Doppler ultrasound sonography of ovarian stromal peak systolic velocity following pituitary suppression can predict COH response more accurately in patients whose FSH levels are normal.

Indications for ovarian reserve tests

Many women face the problem of early reduction of ovarian reserve, affecting their fertility and ability to get pregnant. Nearly 10% of women have early oocyte depletion. Moreover, nowadays, when women prefer late pregnancy, this is a concerning problem as more women will have POR [27]. Hence, women should undergo ORT to plan or modify their pregnancy, and according to their reports, ORT will help them make their reproductive decisions and also help them know about their menopausal timing and reproductive lifespan, etc. ORTs also benefit young cancer patients who may have to go through gonadotoxic therapies.

There are still some questions that need to be addressed about whether or not ORTs should be used for the general population. There are some advantages of using ORTs for the general population as they can help screen patients at risk of early diminution of ovarian reserve. Young women who are at risk of losing their reproductive potential may be identified by this tool, and they can be advised to make family planning a priority [28].

Social egg freezing

It is certain that after the age of 30 years, human eggs start declining in quantity and quality, a trend that drops further down after the age of 35 years. Social egg freezing can help women as it provides insurance to all the women who want to start their families late owing to many reasons related to careers, finances, education, etc. In women in their late thirties, pregnancy gets complicated, and there is a high risk of miscarriage. There is a low fecundity rate and a high risk of pregnancy complications. Even women who are going through gonadotoxic therapies can get their eggs frozen before the therapies so they can consider the idea of pregnancy when they are ready.

Poor ovarian responders

In terms of pregnancy, the poor responders represent a diverse community. Age and oocyte yield significantly impact these patients' chances of getting pregnant [29]. There are no clear guidelines in recent studies on managing POR. Recent studies have shown that various interventions are used to compare the outcomes of ongoing clinical trials. On comparing these interventions, we concluded that any particular intervention would be insufficient [30].

Management

Managing DOR and POR are challenging despite several significant studies and strategies. The objective is to increase the number of oocytes. Various protocols have been suggested for managing patients with DOR and POR. These include estrogen priming, supplementation with LH, high dose of gonadotropins, luteal antagonists, oocyte donation, etc. Various protocols are discussed below.

High-dose Gonadotropins

High-dose gonadotropins are beneficial up to a specific dose, which causes side effects such as increasing the possibility of poor-quality oocytes and patient discomfort. A retrospective study has shown that daily dose and total dose of gonadotropin are inversely related to oocyte production, implantation, live birth rates, and clinical pregnancy. Whether gonadotropin dosage should be increased over 450 IU daily or 3000 IU each cycle is debatable. Excessive stimulation also adversely affects the luteal endocrine milieu, influencing endometrial receptivity [31].

Agonist and Antagonist Protocols

Recent studies have shown that patients treated with contraceptive pills and GnRH-agonists protocol and those treated with GnRH-antagonists protocol have the same outcomes in terms of enhanced pregnancy rates and implantation due to an increased number of retrieved embryos [32].

Several studies have been conducted on using GnRH analog protocol in women with DOR. This protocol can suppress ovaries in women with DOR, further reducing the follicular response. GnRH antagonists are more patient-friendly as they reduce the amount of gonadotropin stimulation. This protocol raises the FSH level between two cycles; hence, less gonadotropin is required. This is why the cycles are relatively shorter, and there is less chance of hypoestrogenic side effects. Recent studies have shown promising results when a combination of corifollitropin alfa with hp-HMG in a GnRH antagonist protocol is used in young women with POR.

Combined Treatment With Letrozole and Clomiphene Citrate 

A study compared the standard high-dose gonadotropin-antagonist regimen with a minimum stimulation protocol overlapping with letrozole in the antagonist cycle in poor responders. It concluded that minimal stimulation IVF protocol results in a higher probability of pregnancy and increased live birth rates [33]. This protocol is also relatively cheaper. Since one experiment included letrozole and the other did not, the outcomes may have been influenced by letrozole usage rather than high vs. low gonadotropin doses, but this cannot be ruled out.

Minimal Stimulation

Recent studies have shown that high-dose gonadotropins are not necessarily required in patients with DOR. Further research has been done to see the difference between the conventional agonist protocol and the moderate gonadotropin protocol with clomiphene citrate and GnRH antagonists comparing live births and pregnancy rates. Concerning clinical pregnancy and live birth rates, this research found that using GnRH antagonists and clomiphene citrate combined with a mild gonadotropin regimen resulted in identical outcomes to conventional stimulation protocol [34].

Use of Adjuvants

Adjuvants are used in the treatment of poor responder patients. Dehydroepiandrosterone (DHEA) and testosterone (T) are also often utilized as adjuvants. Pregnancy rates and oocyte yield increase due to a favorable effect on follicular response to gonadotropin stimulation. Pre-treatment with DHEA and T has been demonstrated to boost live birth rates in women who are poor ovarian responders.

Luteal Estradiol Priming

Estradiol priming in the luteal phase of the menstrual cycle improves follicle synchronization. Studies have shown that estradiol priming during the luteal phase, whether done with or without continuous GnRH antagonist usage, increased the likelihood of conceiving and lowered the risk of cycle cancellation [35].

Recombinant Luteinizing Hormone

In order to produce estrogen and follicles, the female hormones LH and FSH are both required. Recent meta-analyses have shown better chances of pregnancy and increased live birth rates when patients were given LH along with FSH treatment rather than FSH treatment alone.

LH promotes follicle development by assisting in follicle maturation, fertilization, and embryo quality. It has an effect on the endometrium by encouraging the decidualization of endometrial stromal cells and the implantation of embryos. The injection of recombinant human LH (r-hLH) may enhance androgenic and estrogenic follicular fluid levels, which are often reduced in women of late reproductive age. Women beyond the age of 40 years have a decreased chance of having a live birth than women between the ages of 35 and 39 years. Recent studies have revealed that embryo euploidy rates, which are the most important factor related to live births after ART, are significantly higher in women aged 35-39 years than in those over the age of 40 years, a finding that previous studies had failed to reveal because they had not included narrow age ranges.

Several studies have found that recombinant LH and FSH medication enhanced implantation rates and the frequency of positive pregnancy tests in women who had previously failed to conceive. This impact seems to be mostly related to enhanced oocyte quality and the anti-apoptotic action of LH on cumulus cells. Furthermore, LH promotes cell growth and oocyte maturation during folliculogenesis through post-receptor paracrine signaling. This is why recombinant LH and FSH treatment is preferred these days.

Growth Hormone

Growth hormone (GH) modulates the FSH action by regulating insulin production. Several clinical trials have been conducted to see the effect of GH on poor responders. All the studies have shown reduced cancellation rates, increased pregnancy rates, and live birth rates in POR patients who were given GH.

Melatonin

It is believed that taking melatonin before IVF treatment will help patients with PCOS and those with DOR to have better IVF outcomes [36]. It also increases the average estradiol level, mature oocyte counts, and the quality of embryos in patients, leading to better pregnancy and live birth rates.

Oocyte Donation

For patients with DOR and those with POR, egg donation can be their best and final option. Although women who use egg donation have pregnancy rates at least as high as regular responders, the choice is debatable as the facilities and counseling for egg donation are not available to everyone.

## Conclusions

Despite many studies on these topics, we still have not reached a consensus as to how to define and treat reduced ovarian reserve and inadequate ovarian response. The numerous available treatment techniques include increasing the dosage of gonadotropins, flare-up agonists, and gonadotropin-releasing hormone antagonist protocol supplementing with growth hormone, clomiphene citrate, letrozole, androgens, and aspirin. To achieve the best possible reproductive results in such individuals, careful patient counseling and protocol tailoring are essential. Women with DOR need to receive proper counseling on taking an active approach to getting pregnant before it is too late.
